# Case Report: Pulmonary tuberculosis and raised transaminases without pre-existing liver disease- Do we need to modify the antitubercular therapy?

**DOI:** 10.12688/wellcomeopenres.16175.2

**Published:** 2020-10-16

**Authors:** Sanjeev Gautam, Keshav Raj Sigdel, Sudeep Adhikari, Buddha Basnyat, Buddhi Paudyal, Jiwan Poudel, Ujjwol Risal

**Affiliations:** 1 Internal Medicine, Patan Academy of Health Sciences, Lalitpur, State 3, Nepal; 2 Oxford University Clinical Research Unit, Patan Hospital, Lalitpur, State 3, Nepal

**Keywords:** tuberculosis, transaminitis, standard ATT, liver friendly regimen

## Abstract

We report a case of an adult female with pulmonary tuberculosis who had biochemical evidence of liver injury during the presentation manifested as raised transaminases, but without clinically obvious pre-existing liver disease nor a history of hepatotoxic drug use. This is a fairly common scenario seen in tuberculosis endemic areas; however, this is an under reported condition in the literature and guidelines for its management has not been established. Many clinicians including the authors have treated such cases with modified liver friendly regimens in fear of increasing the hepatotoxicity with standard antitubercular drugs. However, the modified regimens may not be optimal in treating the underlying tuberculosis. In this report, we gave full dose standard drugs, and the liver injury resolved as evidenced by normalization of transaminases. Further research is required in this regard, but the presence of transaminitis with no obvious common underlying etiology may not warrant a modification of standard antitubercular regimen.

## Background

Tuberculosis is the biggest infectious disease killer in the world ^1^, and is endemic in Nepal with the national prevalence at 416 cases per 100000 population ^2^. Pulmonary tuberculosis is the most common form. In Nepal, tuberculosis prevalence is more in productive age group (25–64 years) and men. Poverty, malnutrition, overcrowding, immunocompromised state like HIV infection, alcohol, smoking, air pollution, diabetes and other comorbidities are important risk factors for acquiring the disease ^3^. Though under-reported, involvement of liver with tuberculosis is encountered often in clinical practice in endemic areas like Nepal.Liver can be involved; a) diffusely as a part of disseminated miliary tuberculosis or as primary miliary tuberculosis of liver, or b) focal involvement as hepatic tuberculoma or abscess, as was classified by Reed in 1990 ^4^. The biochemical pattern of liver function abnormality in these forms of extrapulmonary tuberculosis is cholestatic (predominantly raised alkaline phosphatase and gamma-glutamyltranspeptidase) rather than hepatocellular (predominantly raised transaminases) ^5, 6^. The hepatocellular pattern of liver injury is seen in cases with pre-existing liver disease including hepatotoxic drug use, which are unrelated to tuberculosis ^7, 8^.

As per national protocol of Nepal, any patient with tuberculosis receives combination antitubercular therapy (ATT) including four drugs; Isoniazid (H), Rifampicin (R), Pyrazinamide (Z) and Ethambutol (E) for initial 2 months popularly known as HRZE. This is followed by 4 months of two drugs; HR. Treatment is given under Directly Observed Treatment Short- Course (DOTS) to improve the patient compliance which could otherwise be compromised owing to lower socioeconomic status of patients, longer duration of treatment and side effects ^9^. Patients with extrapulmonary hepatic tuberculosis are treated with full dose of standard ATT ^5, 6^. But three out of the four drugs (H, R and Z) are hepatotoxic ^7^. So the patients having pre-existing liver disease usually require liver-friendly modified regimens to protect the liver but they may be suboptimal for eradicating underlying tuberculosis ^8^. The protocol of Nepal does not warrant baseline investigations except chest X-ray and sputum smear microscopy to be done routinely before prescribing ATT in programmatic setting ^9^. However in hospital setting like our case, baseline blood investigations including liver function tests are usually done before starting treatment even in absence of features suggesting liver injury and therapy modified accordingly.

Here we present a case of pulmonary tuberculosis with predominant transaminitis but there was no feature of pre-existing liver disease nor a history of hepatotoxic drug use. The liver injury was attributed to the pulmonary tuberculosis itself, and treated with standard first line ATT which led to resolution of liver function abnormalities.

## Case presentation

A 33 year old Newar housewife from Kathmandu, Nepal, with no known comorbidity, presented to Patan Hospital Emergency Department in November, 2019 with a history of cough with occasional sputum production over the previous 20 days and low grade fever for 10 days. There was no history of chest pain, difficulty breathing, headache, vomiting, altered mentation, abdominal pain, yellowish discoloration of eyes, burning urine, hair loss, photosensitivity, joint pain, or rash but she had decreased appetite and weight loss. There was no past history of tuberculosis or jaundice. She did not consume alcohol or any drugs including acetaminophen, aflatoxin or herbal products. Her father-in-law had been diagnosed with pulmonary tuberculosis five years earlier, but there was no family history of liver disease.

Initial examination showed temperature of 101 ^o^F with pulse of 110 beats/minute and respiratory rate of 26 breaths/minute. There was diffuse fine crepitation on the left side on auscultation of the chest. There was no lymphadenopathy, icterus, peripheral edema or wheezes. Neck veins were not distended. Liver and spleen were not palpable, and abdomen examination was normal.

Laboratory parameters with normal ranges in parenthesis are as follow:

Complete blood count before transfusion: white cell count 7.8 (4–10) × 10 ^9^/L; neutrophils 80%; lymphocytes 16%; monocytes 4%; red blood cells 3.6 (4.2–5.4) × 10 ^12^/L; haemoglobin 10.6 (12–15) g/dL; platelets 410 (150–400) × 10 ^9^/L.

Biochemistry: random blood sugar 126 (65–110) mg/dL; urea 39 (17–45) mg/dL; creatinine 1.1 (0.8–1.3) mg/dL; sodium 138 (135–145) mmol/L and potassium 4 (3.5–5) mmol/L.

**Figure 1.**
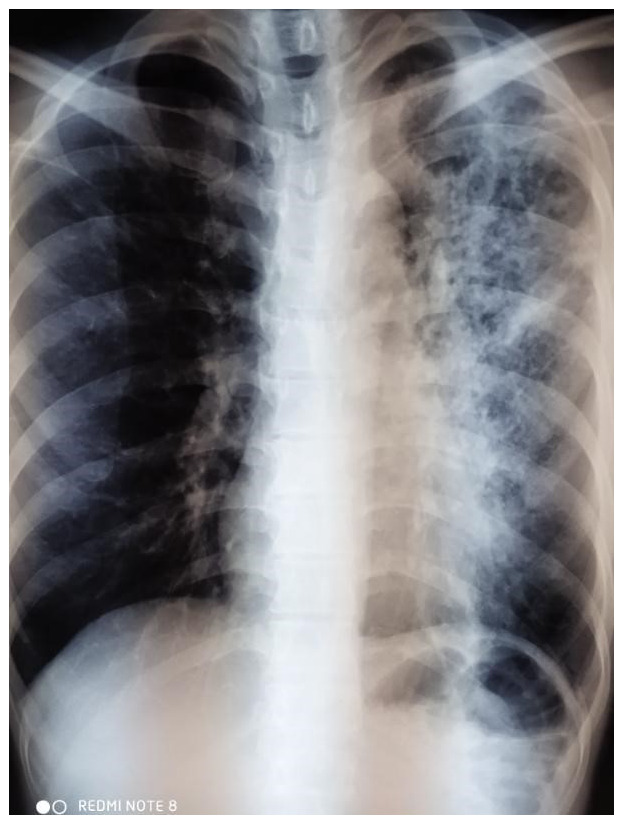
Chest X-ray showing thick walled cavitating lesions in the left upper lobe and patchy infiltrates in left middle and lower zones.

Chest X-ray ( Figure 1) showed thick walled cavitating lesions in the left upper lobe and patchy infiltrates in left middle and lower zones. There were hyperinflated lung fields with blunting of left costophrenic angle. Sputum smear examination showed 3+ acid fast bacilli. Sputum Gene Xpert was positive for Rifampicin sensitive tubercle bacilli. A diagnosis of pulmonary tuberculosis was made, and planned for starting ATT.

Liver function test was performed as baseline workup before starting treatment which showed the following results (with normal ranges in parenthesis): bilirubin total 1.1 (0.1–1.2) mg/dL and direct 0.5 (0–0.4) mg/dL; alanine transaminase 308 (5–30) units/L; aspartate transaminase 605 (5–30) units/L; alkaline phosphatase 149 (50–100) IU/L; gamma-glutamyltranspeptidase66 (9–48) units/L. The raised transaminases led us to perform further workup for liver disease. There was no clinical evidence of chronic liver disease or portal hypertension. Liver synthetic functions were as following;albumin 3.5 (3.5–5) g/dL; total protein 6.5 (6–8.3) g/dL; prothrombin time 14 (11–13.5) s. Serologies for HIV, HBsAg, Hepatitis C virus (HCV), Hepatitis A virus (HAV) and Hepatitis E virus (HEV) were nonreactive. Testing for other hepatotropic viruses was not done because of unavailability of the tests. Neurological examinations and the slit lamp examination of eye were normal. Ultrasound of the abdomen showed a normal sized liver with smooth outline and echotexture. However fibroscan, upper gastrointestinal endoscopy, abdominal CT scan and liver biopsy were not done due to financial constraints of the patient.

She was admitted to the respiratory isolation unit. At first there was some hesitation in starting the full treatment for her pulmonary tuberculosis because of her liver function tests. But taking into consideration her presentation and laboratory findings, we opted for the full treatment rather than a modified TB regimen. We started standard four drugs ATT based on her weight as per national TB guidelines which included three tablets of HRZE given once daily with each tablet containing 75 mg isoniazid (H), 150 mg rifampicin (R), 400 mg pyrazinamide (Z) and 275 mg ethambutol (E). This led to improvement in her clinical status. She was closely observed for possible worsening of her liver disease due to the hepatotoxic antitubercular drugs. Providentially, at 1 week after starting treatment, she was afebrile and continuing to improve and her liver function test showed a total bilirubin of 0.7 mg/dl, aspartate transaminase of 40 IU/L and alanine transaminase of 62 IU/L.

She was discharged with advice to follow up in 1 month. At 1 month follow up she had no symptoms and therefore no further tests were done. At 2 months, she was still asymptomatic and her sputum smear was negative for acid fast bacilli. Her liver function test showed a total bilirubin of 0.6 mg/dl, aspartate transaminase of 30 IU/L and alanine transaminase of 35 IU/L. She was switched to3 tablets of HR to be taken for 4 months.

## Discussion

Our patient with pulmonary tuberculosis had predominantly raised transaminases (hepatocellular pattern)during the initial presentation, with only modest elevation in alkaline phosphatase and gamma glutamyltranspeptidase. The workup for liver disease could not be performed completely because of resource limitation. Looking for clinical evidences by history and examination, and performing liver function tests, abdominal ultrasound and serology for common hepatotropic viruses are usually considered sufficient in our limited setup. We perform further tests only if the initial workup hints towards another etiology. There were no clinical features of chronic liver disease or portal hypertension. She had no risk factors for liver disease such as family history, alcohol, drugs, toxins, features suggesting autoimmune or metabolic liver diseases. Her viral hepatitis serologies were negative. Ultrasound also showed normal liver architecture and size.Though incomplete, the initial workup led us to believe that she had no pre-existing liver injury.

Patients with extrapulmonary hepatic tuberculosis as classified by Reed (diffuse or focal)usually present withnonspecific symptoms like abdominal pain, jaundice, fever, night sweats, fatigue, weight loss and hepatomegaly. They havecholestatic pattern of liver function abnormality with normal transaminases, increased protein- albumin gap owing to raised serum globulin. Hepatic imaging with ultrasound or CT scan reveal abnormalities in 76 and 88% cases respectively. Liver biopsy and demonstration of caseating granuloma and mycobacterial culture remain gold standard for diagnosing hepatic tuberculosis ^4– 6^. Following points in our patient precluded making the diagnosis of hepatic tuberculosis; a) absence of abdominal symptoms and hepatomegaly; b) predominantly raised transaminases (hepatocellular pattern) and normal protein- albumin gap; and c) normal ultrasound finding (though CT and biopsy were not done).

There is another classification schema, given by Levine in 1990 which has incorporated additional entity under hepatic tuberculosis which is ‘pulmonary tuberculosis withliver involvement’ ^10^. In the absence of obvious pre-existing liver disease or drug and the presence of active cavitary tuberculosis in lungs, we attributed the transaminitisin our patient to the pulmonary tuberculosis itself. In our anecdotal experience, we have found many such patients though we do not have any formal data to back this up. They are often managed with modified liver-friendly antitubercular regimens for fear of increasing the hepatotoxicity and causing acute liver failure with the use of standard regimen. Few case reports are available in literature reporting the use of the modified regimens ^11, 12^. We believe such cases are underreported, and firm guidelines have not been established to guide clinicians in these cases. Given this, many clinicians in low-middle income countries, including Nepal, who have been treating tuberculosis patients tend to be skeptical in using full doses of first line ATT in such patients and tend to use a modified regimen. However, this practice may potentially lead to under-treatment and therefore increase fatality ^13^. The use of modified regimen may also increase the risk of developing drug-resistant tuberculosis because of exclusion of more potent drugs ^14^. Though there was some hesitation at first in our case, we soon started treatment with the standard ATT in our patient with close monitoring. This we believe led to the resolution of liver injury, evidenced by the normalization of transaminases.

However, acknowledging that the patient may develop drug induced liver injury (DILI) with the hepatotoxic antitubercular drugs, we should monitorsuch patients closely in an inpatient basis to look forclinical deterioration or any feature suggesting liver failureand liver function test repeated regularly. Though there is no firm recommendation for when to repeat the tests, patient should not be discharged till there is significant improvement in the transaminases level. The close monitoring is important in those with higher risks for developing DILI associated with ATT such as elderly, females, alcohol consumers, the malnourished and those with genetic susceptibility like slow acetylators ^7^. Such monitoring is even more important in our setup because there are possibilities of missing occult hepatic diseases owing tolimited workup.Our patient had improving transaminases evidenced till 2 months follow up.

Though limited by incomplete investigations, we concluded pulmonary tuberculosis as the cause for transaminitis in our patient, and the normalization of transaminases after starting the standard dose of ATT further supports this conclusion. We believe pulmonary TB presenting with transaminitis is a common problem and that treatment may often be compromised because of decreased dosing of ATT.We further aim to perform case series study to explore the magnitude of problem and reach specific conclusions.

## Conclusion

When treating a tuberculosis patient with transaminitis, it is important to look for any possibility of pre-existing liver disease or drug use. If none is found, then the use of standard ATT from the beginning with close inpatient monitoring of the patient may be essential for optimal management of tuberculosis, and this may help resolve any liver injury caused by the tuberculosis. This is a single case report, so further case series or cohort studies would be helpful to reach some conclusion and provide concrete recommendations.

## Consent

Written informed consent for publication of their clinical details and clinical images was obtained from the patient.

## Data availability

### Underlying data

All data underlying the results are available as part of the article and no additional source data are required.
