# Supplementary material for: Case Report: Pulmonary tuberculosis and raised transaminases without pre-existing liver disease- Do we need to modify the antitubercular therapy?
Source: Wellcome Open Res. 2020 Oct 16;5:193. Originally published 2020 Aug 17. [Version 2] doi: 10.12688/wellcomeopenres.16175.2 (PMC7569747; doi:10.12688/wellcomeopenres.16175.2)
Supplement: Peer review history

# Peer review: Reviewer response for version 2

**DOI:** 10.21956/wellcomeopenres.18014.r40969

**Published:** 2020-10-26

**Authors:** Neesha Rockwood

**Affiliations:** 1 Department of Infectious Diseases, Imperial College London, London, UK; 2 Institute of Infectious Diseases and Molecular Medicine, Wellcome Centre for Infectious Diseases Research in Africa, University of Cape Town, Cape Town, South Africa; 3 Department of Microbiology, University of Colombo, Colombu, Sri Lanka

The revised version has been significantly improved. However, further revisions should be made to strengthen the manuscript.

 The statement in the introduction  ‘The liver injury was attributed to the pulmonary tuberculosis itself’ is ambiguous as it is accepted that a significant proportion of patients with pulmonary TB also have extrapulmonary disease. Without a full complement of tests e.g. liver biopsy, CT abdomen, extrapulmonary TB cannot be ruled out.

  In the discussion the authors say ‘Following points in our patient precluded making the diagnosis of hepatic tuberculosis; a) absence of abdominal symptoms and hepatomegaly; b) predominantly raised transaminases (hepatocellular pattern)’. Is diffuse hepatic involvement as a part of disseminated miliary tuberculosis (mentioned in introduction) not compatible with absence of abdominal symptoms and signs and a predominantly transaminitis pattern?

  Please give a table of serial ALT, AST, GGT, Alk phos, Albumin and clotting during inpatient admission to assess trend during early treatment.

  The authors should review the literature for cases where standard quadruple therapy has been utilized for TB with hepatic involvement  and should comment on circumstances when prolongation of length of therapy should be considered.

Are enough details provided of any physical examination and diagnostic tests, treatment given and outcomes?

Partly

Is the case presented with sufficient detail to be useful for other practitioners?

No

Is sufficient discussion included of the importance of the findings and their relevance to future understanding of disease processes, diagnosis or treatment?

Partly

Is the background of the case’s history and progression described in sufficient detail?

Partly

Reviewer Expertise:

Tuberculosis, Interventional Pulmonology, ILD

I confirm that I have read this submission and believe that I have an appropriate level of expertise to confirm that it is of an acceptable scientific standard, however I have significant reservations, as outlined above.

# Peer review: Reviewer response for version 2

**DOI:** 10.21956/wellcomeopenres.18014.r40968

**Published:** 2020-10-16

**Authors:** Vivek Neelakantan

**Affiliations:** 1 Independent Medical Historian, Mumbai, India

The authors have seriously taken note of the reviewer suggestion. The title and the abstract are much clearer. The study leads to an inevitable conclusion. I think the manuscript's status should be emended to indexed. 

Are enough details provided of any physical examination and diagnostic tests, treatment given and outcomes?

Yes

Is the case presented with sufficient detail to be useful for other practitioners?

No

Is sufficient discussion included of the importance of the findings and their relevance to future understanding of disease processes, diagnosis or treatment?

Yes

Is the background of the case’s history and progression described in sufficient detail?

No

Reviewer Expertise:

History of Tuberculosis, Global Health, South and Southeast Asia, Medical Humanities

I confirm that I have read this submission and believe that I have an appropriate level of expertise to confirm that it is of an acceptable scientific standard.

# Peer review: Reviewer response for version 1

**DOI:** 10.21956/wellcomeopenres.17759.r40504

**Published:** 2020-09-24

**Authors:** Neesha Rockwood

**Affiliations:** 1 Department of Infectious Diseases, Imperial College London, London, UK; 2 Institute of Infectious Diseases and Molecular Medicine, Wellcome Centre for Infectious Diseases Research in Africa, University of Cape Town, Cape Town, South Africa; 3 Department of Microbiology, University of Colombo, Colombu, Sri Lanka

Title

 The title needs to convey that this is new onset transaminitis attributed to tuberculosis prior to commencement of anti tubercular therapy

Background

 Please outline the baseline work up for TB in programmatic setting in Nepal. Do all patients have baseline LFTs done?

Case presentation

 Has this patient had normal LFTs recorded prior to current TB presentation?

 Mention drug history incl. paracetamol, aflatoxin exposure, family history of liver disease.

 The liver screen is incomplete - e.g. autoimmune and inherited liver disease, paracetamol levels, ferritin, occult hep B. Also liver fibrosis assessment.  

 Baseline GGT and clotting needs to be given. 

 Please give a table of ALT, AST, GGT, Alk phos, Albumin and clotting during first 2 weeks of treatment (and any further tests during 6 months of treatment)

 Of note, there is no clear evidence of disseminated miliary TB on CXR. Abdominal or liver CT was not done. Hence, no comment can be made regarding  liver/splenic micronodular abscesses. It appears no mycobacterial blood cultures were taken to assess for bacteraemia. Nor was a liver biopsy done. All relevant negatives and limitations should be mentioned.

 When was she discharged? Considering she was never symptomatic from the point of view of GI/hepatobiliary system, was there no further blood work to monitor LFTs since discharge?

Discussion

 Briefly explain classifications of liver TB

 e.g. Reed, Alvarez, Levine.

 The authors do not clearly say what clinical, biochemical, radiological and histopathological presentation would be seen with disseminated TB involving liver.

 This case does not clearly illustrate steps to confirm a transaminitis secondary disseminated miliary TB.

 The implications of this case report would have greater impact and relevance for practitioners in the context of a case series or retrospective cohort ^1 ^and the authors should consider doing this.

 Further discussion is needed of considerations such as potentiated toxicity with pharmacogenomic factors e.g. slow acetylators, the need for individualized monitoring e.g. therapeutic drug monitoring.

 How long should these patients have monitoring of their LFTs? Is there are risk of paradoxical reactions?

Reference

 European Respiratory Journal 2018 52: Suppl. 62, PA4771 ^1^.

Are enough details provided of any physical examination and diagnostic tests, treatment given and outcomes?

Partly

Is the case presented with sufficient detail to be useful for other practitioners?

No

Is sufficient discussion included of the importance of the findings and their relevance to future understanding of disease processes, diagnosis or treatment?

Partly

Is the background of the case’s history and progression described in sufficient detail?

Partly

Reviewer Expertise:

Management of HIV/tuberculosis; PK/PD for tuberculosis

I confirm that I have read this submission and believe that I have an appropriate level of expertise to confirm that it is of an acceptable scientific standard, however I have significant reservations, as outlined above.

## References

[1] Hepatic tuberculosis – a case series. *European Respiratory Journal*.2018; 10.1183/13993003.congress-2018.PA4771 10.1183/13993003.congress-2018.PA4771 Reference source

# Response

**Published:** 2020-10-10

**Authors:** Sudeep Adhikari

**Affiliations:** Patan Academy of Health Sciences, Nepal

We would like to whole-heartedly thank you for spending so much time and effort to improve our manuscript. Here below is the point by point response.

**The title needs to convey that this is new onset transaminitis attributed to tuberculosis prior to commencement of anti tubercular therapy**

Response: Title has been modified. Thank you

**Background Please outline the baseline work up for TB in programmatic setting in Nepal. Do all patients have baseline LFTs done?**

Response: Programmatic setting does not require any baseline workup except sputum smear and in chest Xray. But in hospital setting, we perform baseline blood investigations like blood counts, renal function, electrolytes and liver function tests before starting treatment, so that treatment can be modified accordingly. Changes have been made in the background section.  

**Case presentation**

**Has this patient had normal LFTs recorded prior to current TB presentation?**

Response: No prior LFT testing was done by the patient

**Mention drug history incl. paracetamol, aflatoxin exposure, family history of liver disease.**

Text has been modified to state the fact. Thank you

**The liver screen is incomplete - e.g. autoimmune and inherited liver disease, paracetamol levels, ferritin, occult hep B. Also liver fibrosis assessment.   Baseline GGT and clotting needs to be given.  Please give a table of ALT, AST, GGT, Alk phos, Albumin and clotting during first 2 weeks of treatment (and any further tests during 6 months of treatment) Of note, there is no clear evidence of disseminated miliary TB on CXR. Abdominal or liver CT was not done. Hence, no comment can be made regarding  liver/splenic micronodular abscesses. It appears no mycobacterial blood cultures were taken to assess for bacteraemia. Nor was a liver biopsy done. All relevant negatives and limitations should be mentioned.**

Response: The liver disease screen is incomplete as the reviewer pointed out. However, because of resource limitation and financial constraints, it is not usually possible to perform full liver disease screening in Nepal. So we usually opt for limited screen, and rely more on history, physical examination and initial limited investigations. Then we perform further tests only if the initial workup hints towards another etiology. Text has been modified to include investigations and other limitations. 

**When was she discharged?**

Response: She was discharged after 1 week after becoming afebrile and improvement in transaminases.

**Considering she was never symptomatic from the point of view of GI/hepatobiliary system, was there no further blood work to monitor LFTs since discharge?**

Response: LFT was repeated first at 1 week before discharge, then at 2 months follow up after discharge.

**Discussion:  Briefly explain classifications of liver TB e.g. Reed, Alvarez, Levine. The authors do not clearly say what clinical, biochemical, radiological and histopathological presentation would be seen with disseminated TB involving liver. This case does not clearly illustrate steps to confirm a transaminitis secondary disseminated miliary TB.**

Response: Text has been modified to include further discussion. Thank you

**The implications of this case report would have greater impact and relevance for practitioners in the context of a case series or retrospective cohort and the authors should consider doing this.**

Response: Yes we hope further case reports and case series would be published in future, and we look forward to do case series and we have included this notion in the ms. Thank you. 

**Further discussion is needed of considerations such as potentiated toxicity with pharmacogenomic factors e.g. slow acetylators, the need for individualized monitoring e.g. therapeutic drug monitoring.**

Response: Further discussion has been added. Unfortunately such therapeutic drug monitoring is not widely available in Nepal.

**How long should these patients have monitoring of their LFTs? Is there are risk of paradoxical reactions?**

Unlike Drug induced liver injury, there is no specific monitoring protocols for patients described in our case. The monitoring should be done till the deranged tests normalize and patient becomes clinically well. We have not encountered paradoxical reactions so far.

# Peer review: Reviewer response for version 1

**DOI:** 10.21956/wellcomeopenres.17759.r40181

**Published:** 2020-09-15

**Authors:** Ashesh Dhungana, Prajowl Shrestha

**Affiliations:** 1 Department of Medicine, National Academy of Medical Sciences, Bir Hospital, Kathmandu, Nepal

Title: The title is unclear as to whether the transamnitis in the index patient is caused by tuberculosis (as proposed by the authors in the subsequent report) or a consequence of other coexisting condition. The title needs to be rephrased as to impart a message that the authors want to convey

 Abstract:

- The authors describe a patient with pulmonary tuberculosis and raised liver enzymes (Transaminases). They do mention that the patient did not have any preexisting liver disease or drug use, but do not mention how they systematically ruled out other causes of tansaminitis (other non A-E viral hepatitis like GBV, Hep G, EBV,TT virus and other tropical infections).

References: *DOI: 10.5812/hepatmon.18865* ^1^ *; Alter HJ, Bradley DW. Non-A, non-B hepatitis unrelated to the hepatitis C virus (non ABC) Semin Liver Dis 1995; 15:110-120* ^2^; *Current Opinion in Infectious Diseases: October 2002 - Volume 15 - Issue 5 - p 529-534* ^3^ ***.***

- “In this report, we gave full dose standard antitubercular drugs, and the liver injury resolved evidenced by normalization of transaminases”. The authors should make their message more clear to “presence of transamnitis with no obvious common underlying etiology may not warrant a modification of standard antitubercular regimen”

Case report

1. Evaluation of patient: Diagnostic work-up is incomplete and should also include evaluation for degree of hepatocellular injury. Prothrombin time is not mentioned, GGT levels not mentioned, Serum globulin levels not mentioned (hepatic TB has inverted albumin to globulin ration), imaging was limited (only USG performed, CT not done), liver biopsy not performed. More investigations should have been performed to rule out underlying chronic liver diseases: Fibroscan, upper GI endoscopy for Portal HTN)
2. Figure 1: The quality of the image is suboptimal. The entire bony cage is not visible. Right costophrenic angle is not visible. Finding of hyperinflated lung fields and blunting of left CP angle are not described. Did the patient have underlying obstructive airway disease? If so did she also have pulmonary hypertension?. Could hepatic congestion due to RHF explain the raised liver enzymes?
3. Follow up: The patient improved significantly at 1 month follow up. It would be desirable to have a complete follow up of the patient with evaluation of liver enzymes at least once during treatment as patient initially also did not have any liver specific symptoms.

Discussion:

1. The authors argue that the patient did not have underlying liver disease or liver involvement due to tuberculosis as there was no features of Granuloma and cholestatic pattern of liver enzyme elevation. To ascribe the transamnitis to be caused by TB would be an arbitrary statement especially in the absence of liver biopsy.
2. The authors mention that they have found many patients of pulmonary TB to have predominant transamnitis and without any preexisting liver disease in their experience. Such statement is not backed by any formal data.
3. The authors conclude that pulmonary TB was the cause of transamnitis in their patient, not hepatic TB or underlying liver disease. In the absence of complete workup, such strong conclusions should not be made.

Conclusion

 The authors recommend use of full dose standard ATT for patients with transamnitis and no underlying liver disease. This recommendation should not be made based on a single case report.

 Opinion:

 The case report describes a common scenario especially in low income countries while treating patients with tuberculosis. The availability of resources limit the diagnostic workup of such patients in our settings. I opine that this case report is suitable for indexing with modifications.

Are enough details provided of any physical examination and diagnostic tests, treatment given and outcomes?

Partly

Is the case presented with sufficient detail to be useful for other practitioners?

Partly

Is sufficient discussion included of the importance of the findings and their relevance to future understanding of disease processes, diagnosis or treatment?

Partly

Is the background of the case’s history and progression described in sufficient detail?

Yes

Reviewer Expertise:

Tuberculosis, Interventional Pulmonology, ILD

We confirm that we have read this submission and believe that we have an appropriate level of expertise to confirm that it is of an acceptable scientific standard, however we have significant reservations, as outlined above.

## References

[1] : Epstein-Barr Virus Infection Mimicking Drug-Induced Hepatitis in a Critically ill Patient During Antituberculosis Therapy. *Hepatitis Monthly*.2014;14(9) : 10.5812/hepatmon.18865 10.5812/hepatmon.18865 PMC421412225368656

[2] : Non-A, non-B hepatitis unrelated to the hepatitis C virus (non-ABC). *Semin Liver Dis*.1995;15(1) : 10.1055/s-2007-1007268 110-20 10.1055/s-2007-1007268 7597441

[3] : Non-A to E hepatitis. *Current Opinions in Infectious Diseases*.2002;15(5) :529-534 Reference source 10.1097/00001432-200210000-0001312686888

# Response

**Published:** 2020-10-10

**Authors:** Sudeep Adhikari

**Affiliations:** Patan Academy of Health Sciences, Nepal

**We would like to whole-heartedly thank you for spending so much time and effort to improve our manuscript. Here below is the point by point response.**

**Title: The title is unclear as to whether the transaminitis in the index patient is caused by tuberculosis (as proposed by the authors in the subsequent report) or a consequence of other coexisting condition. The title needs to be rephrased as to impart a message that the authors want to convey**

 Response: Title has been modified to make our message clearer. Thank you

**Abstract: The authors describe a patient with pulmonary tuberculosis and raised liver enzymes (Transaminases). They do mention that the patient did not have any preexisting liver disease or drug use, but do not mention how they systematically ruled out other causes of tansaminitis (other non A-E viral hepatitis like GBV, Hep G, EBV,TT virus and other tropical infections). ○ References: DOI: 10.5812/hepatmon.188651; Alter HJ, Bradley DW. Non-A, non-B hepatitis unrelated to the hepatitis C virus (non ABC) Semin Liver Dis 1995; 15:110-1202; Current Opinion in Infectious Diseases: October 2002 - Volume 15 - Issue 5 - p 529-5343.**

 Testing of other viruses were not done due to unavailability.Since raised transaminases resolved after starting ATT we assumed tuberculosis was most likely explanation.

**“In this report, we gave full dose standard antitubercular drugs, and the liver injury resolved evidenced by normalization of transaminases”. The authors should make their message more clear to “presence of transaminitis with no obvious common underlying etiology may not warrant a modification of standard antitubercular regimen”**

 Response: Text has been modified to make our message clearer. Thank you

**Case report Evaluation of patient: Diagnostic work-up is incomplete and should also include evaluation for degree of hepatocellular injury. Prothrombin time is not mentioned, GGT levels not mentioned, Serum globulin levels not mentioned (hepatic TB has inverted albumin to globulin ration), imaging was limited (only USG performed, CT not done), liver biopsy not performed. More investigations should have been performed to rule out underlying chronic liver diseases: Fibroscan, upper GI endoscopy for Portal HTN)**

 Response: The liver disease screen is incomplete as the reviewers pointed out. However, because of resource limitation and financial constraints, it is not usually possible to perform full liver disease screening in Nepal. So we usually opt for limited screen, and rely more on history, physical examination and initial limited investigations. Then we perform further tests only if the initial workup hints towards another etiology. Text has been modified to include further lab reports. The limitations have been acknowledged.

**Figure 1: The quality of the image is suboptimal. The entire bony cage is not visible. Right costophrenic angle is not visible. Finding of hyperinflated lung fields and blunting of left CP angle are not described. Did the patient have underlying obstructive airway disease? If so did she also have pulmonary hypertension?. Could hepatic congestion due to RHF explain the raised liver enzymes?**

 Response: There was no history of underlying lung disease. Although echocardiography was not done hepatic congestion due to RHF is unlikely as there was no suggestive history and examination findings and patient responded without any diuretics or fluid restrictions.

**Follow up: The patient improved significantly at 1 month follow up. It would be desirable to have a complete follow up of the patient with evaluation of liver enzymes at least once during treatment as patient initially also did not have any liver specific symptoms.**

 Response: Text has been modified to include follow up reports. LFT was repeated first at 1 week before discharge, then at 2 months follow up after discharge.

**Discussion:**

**The authors argue that the patient did not have underlying liver disease or liver involvement due to tuberculosis as there was no features of Granuloma and cholestatic pattern of liver enzyme elevation. To ascribe the transaminitis to be caused by TB would be an arbitrary statement especially in the absence of liver biopsy.**

 Response: Imaging and further investigations were limited, so hepatic tuberculosis could not be ruled out with certainty especially without biopsy of liver. However this would not make much difference. Because even if the diagnosis of hepatic tuberculosis had been considered in our patient, the management would be the same, i.e. with standard ATT as we did in our patient. The point we wanted to make here is that modification in treatment may not be required in the absence of pre-existing liver disease. Text has been modified to include the limitations. Thank you

**The authors mention that they have found many patients of pulmonary TB to have predominant transaminitis and without any preexisting liver disease in their experience. Such statement is not backed by any formal data.**

 Response: We need more data but unfortunately not much is being published from low middle income countries even for endemic diseases like tuberculosis. There is no formal data to back up our claim, and this has been acknowledged as limitation.

**The authors conclude that pulmonary TB was the cause of transaminitis in their patient, not hepatic TB or underlying liver disease. In the absence of complete workup, such strong conclusions should not be made.**

 Response: Due to rapid resolution of raised transaminases after starting ATT, preexisting liver disease would be unlikely cause. Although hepatic tb is a possibility, it was our opinion that standard dose ATT can be safely started in the patient and further imaging would not be cost effective in terms of treatment. We agree with the reviewer that the strong conclusions are not justified due to incomplete workup and this section has been toned down and modified to highlight the limitations. Thank you

**Conclusion The authors recommend use of full dose standard ATT for patients with transaminitis and no underlying liver disease. This recommendation should not be made based on a single case report.**

 Response: We agree. Text have been modified to include our limitations. Thank you.

**Opinion: The case report describes a common scenario especially in low income countries while treating patients with tuberculosis. The availability of resources limit the diagnostic workup of such patients in our settings. I opine that this case report is suitable for indexing with modifications.**

 Response: Thank you

# Peer review: Reviewer response for version 1

**DOI:** 10.21956/wellcomeopenres.17759.r39997

**Published:** 2020-08-27

**Authors:** Vivek Neelakantan

**Affiliations:** 1 Independent Medical Historian, Mumbai, India

**Title:**

 Drug-resistant TB is a hot topic in medicine today. Research on TB treatment-associated transaminitis would further the existing scholarly understanding on drug-resistant TB. A close keyword search on PubMed revealed only 17 hit on TB AND transaminitis. For this reason, the  case report is potentially publishable but suffers from several drawbacks in its current draft that must be remedied before the contribution is re-refereed. 

**But **the title itself is unclear. “With” is repeated twice. It is not clear to a medical historian what transaminitis is. A better framing of the title is urgently needed. A cogent argument can be organised around the title. What is four-drugs therapy? It is clear to medical practitioners but not clear to the larger scholarly community. Avoid jargons in the title.

 1)    What is transaminitis? Why not provide a brief explanation at the start so that the general reader can follow the rest of the article.

 2)    The two levels of transaminitis:

 (a) pre-existing liver disease leads to transaminitis.

 (b) Hepatoxicity of anti-TB drugs.

 This needs to be made explicit in the very beginning. The authors have not been explicit about the two levels of transaminitis and that is where the problem begins.

 “While encountering such patients, it is important to differentiate if the patient had pre-existing liver disease or if the present infection with tuberculosis has impacted on the liver, as the approach to management differs given the hepatotoxicity associated with first line drugs.”

 Rewrite this sentence. Make more explicit.

**Summary and Abstract:**

 The writers present a case of pulmonary TB with transaminitis without pre-existing liver damage. The therapeutic regimen of the authors included anti-TB drugs and liver injury resolved evidenced by normalization of transaminase.

 The abstract merits rewriting for clarity. 

**Background:**

  Background: Needs to sketch out the larger socio-economic picture of TB patients in Nepal. Medicine for whom? 

**Case Presentation:**  How do you define compliance with TB treatment? How socio-economic factors militate against the successful completion of treatment?

 Specific quote from the report: “In our anecdotal experience, we have found many patients with pulmonary tuberculosis, similarly to subject of this case report, present with predominant transaminitis and without pre-existing liver disease or drugs-use. They are often managed with modified liver-friendly antitubercular regimens for fear of increasing the hepatotoxicity and causing acute liver failure with the use of standard regimen. Few case reports are available in literature reporting the use of the modified regimens ^7^ ^,^ ^8^. We believe such cases are underreported, and firm guidelines have not been established to guide clinicians in these cases. Given this, many clinicians in low-middle income countries, including Nepal, who have been treating tuberculosis patients tend to be skeptical in using full doses of first line ATT in such patients and tend to use a modified regimen. However, this practice may potentially lead to under-treatment and therefore increase fatality ^9^. Though there was some hesitation at first in our case, we soon started treatment with the standard ATT in our patient with close monitoring. This we believe led to the resolution of liver injury, evidenced by the normalization of transaminases.”

What is your sample size? Unclear. What guidelines can be established with the aid of the study?" Make explicit and elaborate.

**Conclusion:**

 The report lacks an inevitable conclusion. 

 The conclusion needs to point to the “so what” question? So, what are the implications of this study? What protocols could be devised? How does this case history further medical practitioners’ as well as policymakers’ understanding of drug-resistant TB?

**Other points:**

 Please make sure that the manuscript is thoroughly copyedited for legibility of prose, clarity of argument, and grammar. 

 The social context of TB in Nepal merits attention (alcoholism is a contributing factor).

 You need to compare transaminitis with case studies from other countries. Carefully refer to the PLoS

**One article below:**

 Murphy, Richard A., Vincent C. Marconi, Rajesh T. Gandhi, Daniel R. Kuritzkes, and Henry Sunpath. "Coadministration of lopinavir/ritonavir and rifampicin in HIV and tuberculosis co-infected adults in South Africa." *PLoS One* 7, no. 9 (2012): e44793 ^1^.

 Sarda, Pawan, S. K. Sharma, Alladi Mohan, Govind Makharia, Arvind Jayaswal, R. M. Pandey, and Sarman Singh. "Role of acute viral hepatitis as a confounding factor in antituberculosis treatment induced hepatotoxicity." *Indian Journal of Medical Research* 129, no. 1 (2009): 64 ^2^.

 How does your case differ from the 2 references mentioned above?

Are enough details provided of any physical examination and diagnostic tests, treatment given and outcomes?

Yes

Is the case presented with sufficient detail to be useful for other practitioners?

No

Is sufficient discussion included of the importance of the findings and their relevance to future understanding of disease processes, diagnosis or treatment?

Yes

Is the background of the case’s history and progression described in sufficient detail?

No

Reviewer Expertise:

History of Tuberculosis, Global Health, South and Southeast Asia, Medical Humanities

I confirm that I have read this submission and believe that I have an appropriate level of expertise to state that I do not consider it to be of an acceptable scientific standard, for reasons outlined above.

## References

[1] : Correction: Coadministration of Lopinavir/Ritonavir and Rifampicin in HIV and Tuberculosis Co-Infected Adults in South Africa. *PLoS One*.2013;8(12) : 10.1371/annotation/730cdfd0-78c5-48fc-a095-f633905ff2f0 10.1371/annotation/730cdfd0-78c5-48fc-a095-f633905ff2f0 29220839PMC5730193

[2] : Role of acute viral hepatitis as a confounding factor in antituberculosis treatment induced hepatotoxicity. *Indian J Med Res*.2009;129(1) :64-7 19287059

# Response

**Published:** 2020-10-10

**Authors:** Sudeep Adhikari

**Affiliations:** Patan Academy of Health Sciences, Nepal

**We would like to whole-heartedly thank you for spending so much time and effort to improve our manuscript. Here below is the point by point response.**

**Title:  Drug-resistant TB is a hot topic in medicine today. Research on TB treatment-associated transaminitis would further the existing scholarly understanding on drug-resistant TB. A close keyword search on PubMed revealed only 17 hit on TB AND transaminitis. For this reason, the  case report is potentially publishable but suffers from several drawbacks in its current draft that must be remedied before the contribution is re-refereed.  But the title itself is unclear. “With” is repeated twice. It is not clear to a medical historian what transaminitis is. A better framing of the title is urgently needed. A cogent argument can be organised around the title. What is four-drugs therapy? It is clear to medical practitioners but not clear to the larger scholarly community. Avoid jargons in the title. 1)    What is transaminitis? Why not provide a brief explanation at the start so that the general reader can follow the rest of the article. 2)    The two levels of transaminitis: (a) pre-existing liver disease leads to transaminitis. (b) Hepatoxicity of anti-TB drugs. This needs to be made explicit in the very beginning. The authors have not been explicit about the two levels of transaminitis and that is where the problem begins. “While encountering such patients, it is important to differentiate if the patient had pre-existing liver disease or if the present infection with tuberculosis has impacted on the liver, as the approach to management differs given the hepatotoxicity associated with first line drugs.” Rewrite this sentence. Make more explicit.**

**Response: Title and text have been modified to make our message clearer. Thank you**

**Summary and Abstract:  The writers present a case of pulmonary TB with transaminitis without pre-existing liver damage. The therapeutic regimen of the authors included anti-TB drugs and liver injury resolved evidenced by normalization of transaminase. The abstract merits rewriting for clarity.**

**Response: Abstract has been modified to make our message clearer**

**Background:  Needs to sketch out the larger socio-economic picture of TB patients in Nepal. Medicine for whom?**

**Response: Background has been modified to make it clear. The socioeconomic picture of TB patients in Nepal has been highlighted. In Nepal, tuberculosis prevalence is more in productive age group (25-64 years) and men. Poverty, malnutrition, overcrowding, immunocompromised state like HIV infection, alcohol, smoking, air pollution, diabetes and other comorbidities are important risk factors for acquiring the disease. All patients diagnosed with TB receive treatment as per the national protocol which has been mentioned in the text.**

**Case Presentation:**

**How do you define compliance with TB treatment? How socio-economic factors militate against the successful completion of treatment?**

**Response: To improve the treatment compliance, treatment of TB is done under DOTS program all over Nepal, which stands for ‘Directly Observed Treatment Short Course’. Otherwise the compliance would be compromised owing to the lower socioeconomic status of patients, longer duration of therapy and side effects of drugs.**

**Specific quote from the report: “In our anecdotal experience, we have found many patients with pulmonary tuberculosis, similarly to subject of this case report, present with predominant transaminitis and without pre-existing liver disease or drugs-use. They are often managed with modified liver-friendly antitubercular regimens for fear of increasing the hepatotoxicity and causing acute liver failure with the use of standard regimen. Few case reports are available in literature reporting the use of the modified regimens. We believe such cases are underreported, and firm guidelines have not been established to guide clinicians in these cases. Given this, many clinicians in low-middle income countries, including Nepal, who have been treating tuberculosis patients tend to be skeptical in using full doses of first line ATT in such patients and tend to use a modified regimen. However, this practice may potentially lead to undertreatment and therefore increase fatality9. Though there was some hesitation at first in our case, we soon started treatment with the standard ATT in our patient with close monitoring. This we believe led to the resolution of liver injury, evidenced by the normalization of transaminases.” What is your sample size? Unclear. What guidelines can be established with the aid of the study?" Make explicit and elaborate**

**Response: Our opinion is that there are cases of pulmonary tb with some hepatic involvement, which are sometimes being managed with modified regimen when it can be safely managed with standard regimen. Unfortunately very few published research is available from low middle income countries. Text has been edited to include limitation of evidence. This is a case report only and further research is needed.**

**Conclusion:**

**The report lacks an inevitable conclusion.  The conclusion needs to point to the “so what” question? So, what are the implications of this study? What protocols could be devised? How does this case history further medical practitioners’ as well as policymakers’ understanding of drug-resistant TB?**

**Response: Being a case report and paucity of previous research, we have toned down our previous strong conclusions, also as suggested above by another reviewer. We hope that further reports will be published. The use of modified drug regimen excluding more potent drugs, instead of standard regimen may promote drug resistance, and how far such practices are prevalent can be another area of further study.**

**Other points:  Please make sure that the manuscript is thoroughly copyedited for legibility of prose, clarity of argument, and grammar.  The social context of TB in Nepal merits attention (alcoholism is a contributing factor). You need to compare transaminitis with case studies from other countries. Carefully refer to the PLoS**

**Response: Text has been revised to correct errors. Social context of TB in Nepal has been mentioned in the text. Thank you**

**Murphy, Richard A., Vincent C. Marconi, Rajesh T. Gandhi, Daniel R. Kuritzkes, and Henry Sunpath. "Coadministration of lopinavir/ritonavir and rifampicin in HIV and tuberculosis co-infected adults in South Africa." PLoS One 7, no. 9 (2012): e447931. Sarda, Pawan, S. K. Sharma, Alladi Mohan, Govind Makharia, Arvind Jayaswal, R. M. Pandey, and Sarman Singh. "Role of acute viral hepatitis as a confounding factor in antituberculosis treatment induced hepatotoxicity." Indian Journal of Medical Research 129, no. 1 (2009): 642. How does your case differ from the 2 references mentioned above?**

**Response: As compared to first article our patient did not have HIV. As compared to second article, our patient did not have antituberculous drug induced hepatotoxicity but liver injury likely due to tuberculosis itself. Thank you**
